# Transformation from hematoxylin-and-eosin staining to Ki-67 immunohistochemistry digital staining images using deep learning: experimental validation on the labeling index

**DOI:** 10.1117/1.JMI.11.4.047501

**Published:** 2024-07-30

**Authors:** Cunyuan Ji, Kengo Oshima, Takumi Urata, Fumikazu Kimura, Keiko Ishii, Takeshi Uehara, Kenji Suzuki, Saori Takeyama, Masahiro Yamaguchi

**Affiliations:** a Tokyo Institute of Technology, School of Engineering, Department of Information and Communications Engineering, Yokohama, Japan; b Shinshu University, School of Health Sciences, Department of Biomedical Laboratory Sciences, Matsumoto, Japan; c Okaya City Hospital, Division of Diagnostic Pathology, Okaya, Japan; d Shinshu University, School of Medicine, Department of Laboratory Medicine, Matsumoto, Japan; e Tokyo Institute of Technology, Institute of Innovative Research, Biomedical AI Research Unit, Yokohama, Japan

**Keywords:** digital staining, labeling index, whole slide image, deep learning, Ki-67

## Abstract

**Purpose:**

Endometrial cancer (EC) is one of the most common types of cancer affecting women. While the hematoxylin-and-eosin (H&E) staining remains the standard for histological analysis, the immunohistochemistry (IHC) method provides molecular-level visualizations. Our study proposes a digital staining method to generate the hematoxylin-3,3′-diaminobenzidine (H-DAB) IHC stain of Ki-67 for the whole slide image of the EC tumor from its H&E stain counterpart.

**Approach:**

We employed a color unmixing technique to yield stain density maps from the optical density (OD) of the stains and utilized the U-Net for end-to-end inference. The effectiveness of the proposed method was evaluated using the Pearson correlation between the digital and physical stain’s labeling index (LI), a key metric indicating tumor proliferation. Two different cross-validation schemes were designed in our study: intraslide validation and cross-case validation (CCV). In the widely used intraslide scheme, the training and validation sets might include different regions from the same slide. The rigorous CCV validation scheme strictly prohibited any validation slide from contributing to training.

**Results:**

The proposed method yielded a high-resolution digital stain with preserved histological features, indicating a reliable correlation with the physical stain in terms of the Ki-67 LI. In the intraslide scheme, using intraslide patches resulted in a biased accuracy (e.g., R=0.98) significantly higher than that of CCV. The CCV scheme retained a fair correlation (e.g., R=0.66) between the LIs calculated from the digital stain and its physical IHC counterpart. Inferring the OD of the IHC stain from that of the H&E stain enhanced the correlation metric, outperforming that of the baseline model using the RGB space.

**Conclusions:**

Our study revealed that molecule-level insights could be obtained from H&E images using deep learning. Furthermore, the improvement brought via OD inference indicated a possible method for creating more generalizable models for digital staining via per-stain analysis.

## 1 Introduction

Hematoxylin and eosin (H&E) staining is a general staining method commonly performed in pathological diagnosis. Hematoxylin dye stains the cell nuclei, whereas eosin dye stains the cytoplasm. The morphological features, such as cell and tissue structures, morphology, color, and texture, are evaluated to determine the pathological diagnosis. H&E staining is followed by the classification of the histological type and differentiation grade. Various proteins within the cells related to targeted therapy are visualized via immunohistochemical (IHC) reactions. IHC staining has been used to visualize estrogen receptor, progesterone receptor, human epidermal growth factor receptor 2 (HER2), and Ki-67 in patients with breast cancer. In IHC, various proteins are visualized using 3,3′-diaminobenzidine (DAB), and the nuclei are visualized via counterstaining with hematoxylin.^1^ The IHC staining method plays a vital role in the pathology diagnosis of cancers; however, it is more expensive and complicated than H&E staining.

**Fig. 1:**
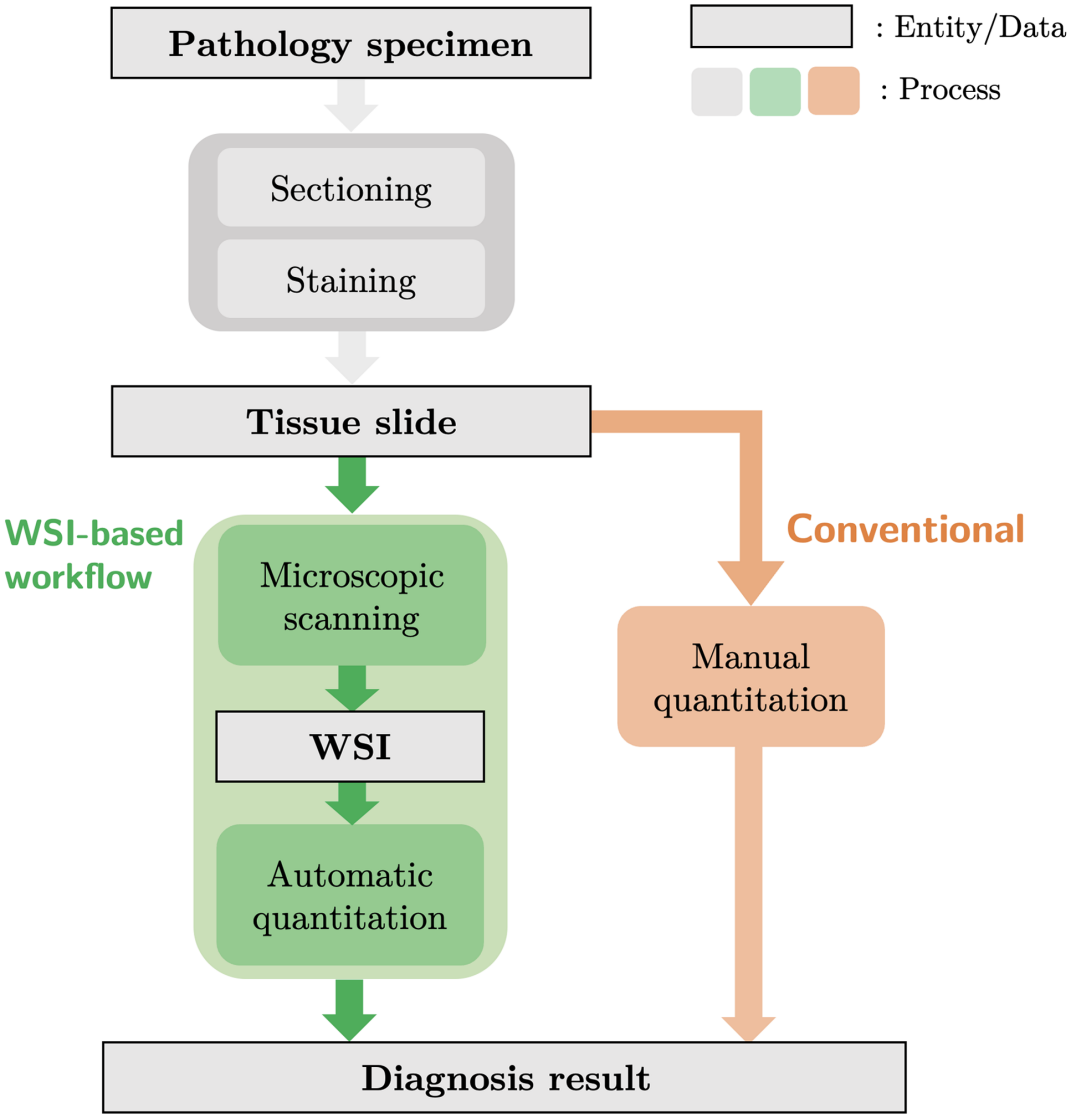
Workflow of pathological diagnosis.

**Fig. 2:**
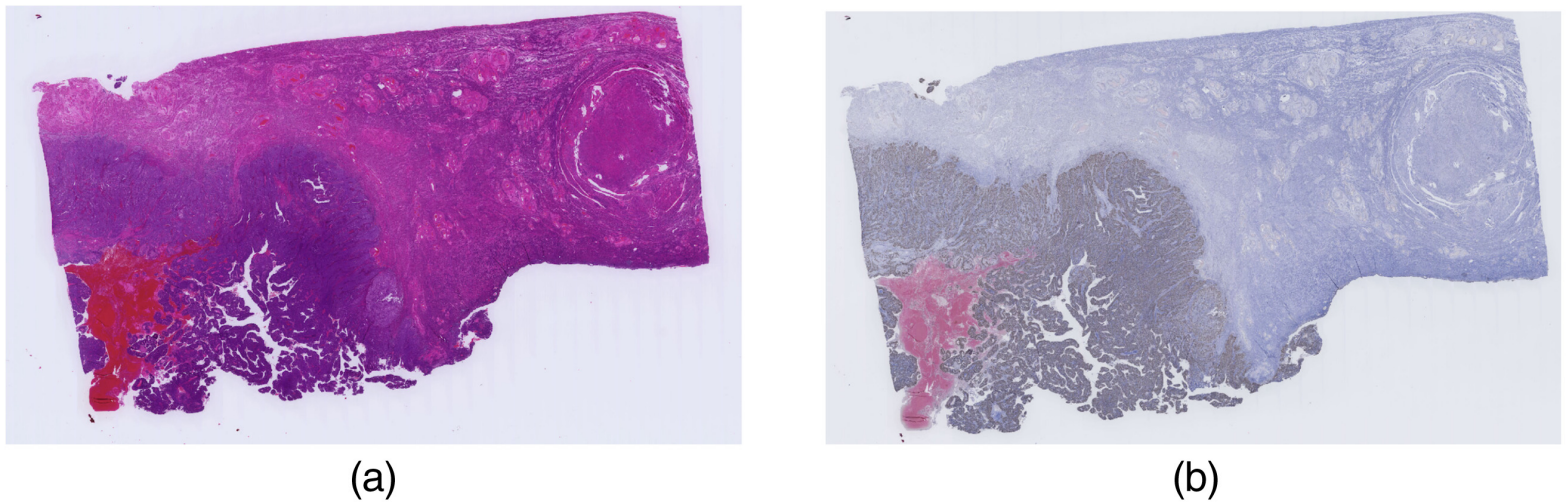
Representative images depicting the (a) H&E and (b) Ki-67 immunohistochemistry stains.

Whole slide imaging (WSI) technology has revolutionized the domain of pathology diagnosis. Tissue slides are digitized into high-resolution images using microscopic scanners (Fig. 1), and the laborious process of manual quantitation is replaced with efficient automated algorithms. Figure 2 presents the H&E stain and IHC stain WSIs of a uterine corpus specimen. Additionally, storing digital images allows the application of image analysis technology, or artificial intelligence, including deep learning technology. Powered by the evolution of computing hardware such as the graphics processing unit (GPU), deep learning technology has achieved impressive outcomes in the field of computer vision. Previous studies have established the ability of deep learning techniques to perform pathology image analysis tasks, such as the classification of histological types and differentiation of cancer, the detection of mitotic cells in the tissues, and the segmentation of the tumors.

Ki-67 protein is expressed during the G1, S, G2, and M phases of the cell cycle, except for the quiescent phase (G0).^1^^,^^2^ Consequently, Ki-67 has been used as a biomarker to assess the proliferative ability of malignant cells and determine the malignancy of cancer.^3^

The labeling index (LI), also known as the proliferation index,^4^ is one of the crucial diagnostic parameters calculated from the IHC expression of Ki-67. The LI represents the ratio between the number of IHC-positive nuclei and the total number of nuclei within the tumor. After obtaining the whole slide images, pathologists would select regions of interest (ROIs) from hotspots containing sufficient expression areas for quantification.

An ROI should contain around 1000 to 1200 cell nuclei for the calculation of LI.^5^ However, manual measurement and evaluation of LI is labor-intensive. Therefore, a technique for converting the H&E-stained specimens into their IHC-stained counterparts, which can be used for automatic quantitation, was developed in this study using deep learning and image processing methods. The Ki-67 protein is expressed during the active phases of the cell cycle. Thus the expression of this histochemical can be inferred from the morphological and texture features visualized via the H&E staining method.^1^^,^^2^^,^^6^^–^^8^ Deep learning methods could facilitate this process. U-Net, a deep learning model for image segmentation, was used in this study to generate digital hematoxylin-3,3′-diaminobenzidine (H-DAB) IHC stains with accurate nuclear positivity. The proposed method was applied to the WSIs of patients with endometrial adenocarcinoma. Previous studies have evaluated the utility of digital IHC staining using deep learning.^9^ However, this is the first study to depict the correlation between the LI of digital IHC staining and physical IHC staining and analyze the cross-case generalization of Ki-67 digital staining models in uterine corpus endometrial carcinoma (UCEC). 

## 2 Related Work

### 2.1 Encoder–Decoder Models

Deep learning methods have been widely used to perform generative computer vision tasks. The encoder–decoder architecture is one such popular paradigm. The encoder accepts an input image and projects it to a high-dimensional feature space with a relatively lower spatial resolution and abundant semantic information. The decoder recovers an output containing task-specific information, such as the segmentation of objects or images with alternated styles,^10^ from the encoded tensors. The fully convolutional neural network^11^ (FCN) was designed for semantic segmentation in general scenes. FCN was the first network to produce a pixel-to-pixel translation of images using convolutional layers only. Compared with its predecessors, FCN contains no dense layer but introduces the upsampling operation to decode the output image with a resolution identical to the input from the feature maps encoded by convolutional layers from the input image. U-Net is a widely used model for segmenting medical images^12^ and generative computer vision tasks. Compared with FCN, U-Net inserts skip connections between the encoder and decoder layers. This operation contributes to aggregating information from different scales and generates fine-grained results.

### 2.2 Classification of Ki-67-Positive Nuclei Using Hand-Crafted Features

Cells in different phases of the cell cycle possess unique morphological and texture features.^13^ Kimura et al.^6^ used the support vector machine (SVM) to classify the Ki-67-positive and Ki-67-negative single nuclei cropped from endometrial adenocarcinoma specimens. The nuclei were extracted and divided into positive and negative groups equally. The signal intensities, texture features represented by the gray-level co-occurrence matrix,^14^ morphological features, and chromatin distributions of each nucleus were differentiated using a linear SVM.^15^ This method resulted in an accuracy of 85%. Those studies suggested that the proliferation status of cells may be correlated with the morphological and texture characteristics. Therefore, it may be possible to translate an H&E-stained specimen to its IHC counterpart by analyzing the features of the nuclei and identifying the proliferating nuclei that should be marked with the DAB component.

### 2.3 Digital Staining

Digital staining has enabled the visualization of tissue regions via the analysis of their features using algorithms instead of physical pigments. Traditionally, digital staining can be realized by analyzing the spectral characteristics of the tissue.^16^ Advances in the field of deep learning have facilitated further research on transforming the stain types with neural networks. By leveraging the visual features of different tissue regions, colors of corresponding pigments are assigned to each tissue area. For example, Chang et al.^17^ proposed to transform H&E to the immunofluorescence stain using the Pix2Pix model,^18^ Xu et al.^19^ and Quiros et al.^20^ used adversarial networks to generate real-like stained specimen samples. De Haan et al.^21^ used GAN-based methods to transfer the H&E stain to Masson’s Trichrome, Jones, and PAS stains. However, these stains are histological stains corresponding to human-visible structures, such as membranes or fibers, and have no functionality to reveal molecule-level activities. Mercan et al.^22^ utilized the Cycle-GAN^23^ to map an image of H&E stained breast specimen to its phosphohistone H3 (pHH3) stain counterpart and revealed the presence of mitotic cells in the tissue. Li et al.^24^ used a U-Net with Gaussian-weighted masks of cell centroids to distinguish the mitotic cells and revealed a correlation between the visual patterns in H&E images and the cell cycle information revealed by pHH3. Notice that their problem setting is similar to the work presented herein, whereas the pHH3 is only expressed during the mitosis and G2 phases, and Ki-67 is expressed during all active phases of the cell cycle.^25^ Moreover, the mitotic cells in the H&E stained specimens can be distinguished visually, whereas Ki-67 positive cells cannot be directly observed. Therefore, utilizing features related to Ki-67 expression is a more challenging task as the visual characteristics are relatively subtle during nonmitotic phases.

Three highly related studies are introduced herein. Liu et al.^26^ used ResNet-15^27^ to classify manually annotated nucleus patches in neuroendocrine tumors. The network was reformed into an FCN to generate a heatmap of positive nuclei. A strong correlation was observed between the positive pixel area ratios in the prediction and the ground truth. Liu et al.^28^ used a Cycle-GAN-like model on serial cuts of neuroendocrine cancer and breast cancer to generate digital Ki-67 stains and obtained a strong correlation of Ki-67 positive area. Martino et al.^29^ used the Pix2Pix model to predict Ki-67 positivity in H&E-stained oral squamous cell carcinoma tissues and reported a strong correlation of the LI.

Precedent research has shown the possibility of stain conversion with generative models. However, the generalization of models has not been elucidated, especially in terms of the reliable derivation of nucleus-level diagnostic metrics in intercase scenarios. Moreover, the results of the nucleus-level evaluation, such as LI, have not been reported, and the cross-case performance of their model remains unclear. Additionally, the FCN sacrifices image resolution as it downsamples the image, whereas a U-Net-based generator can preserve the resolution of the input.

In this study, we used cross-case schemes to quantitatively evaluate the generalization gap of U-Net-based Ki-67 LI prediction across cases. This study presents the results of deriving stain density maps from the optical density (OD) image instead of the RGB image. Compared with the RGB space, using the OD images to train the U-Net improved the correlation of the LI under the intraslide and cross-case scenarios. This is the first report to depict the correlation between the LIs of digital IHC stain and physical stain in a cross-case condition for UCEC.

## 3 Methodology

### 3.1 Overview

**Fig. 3:**
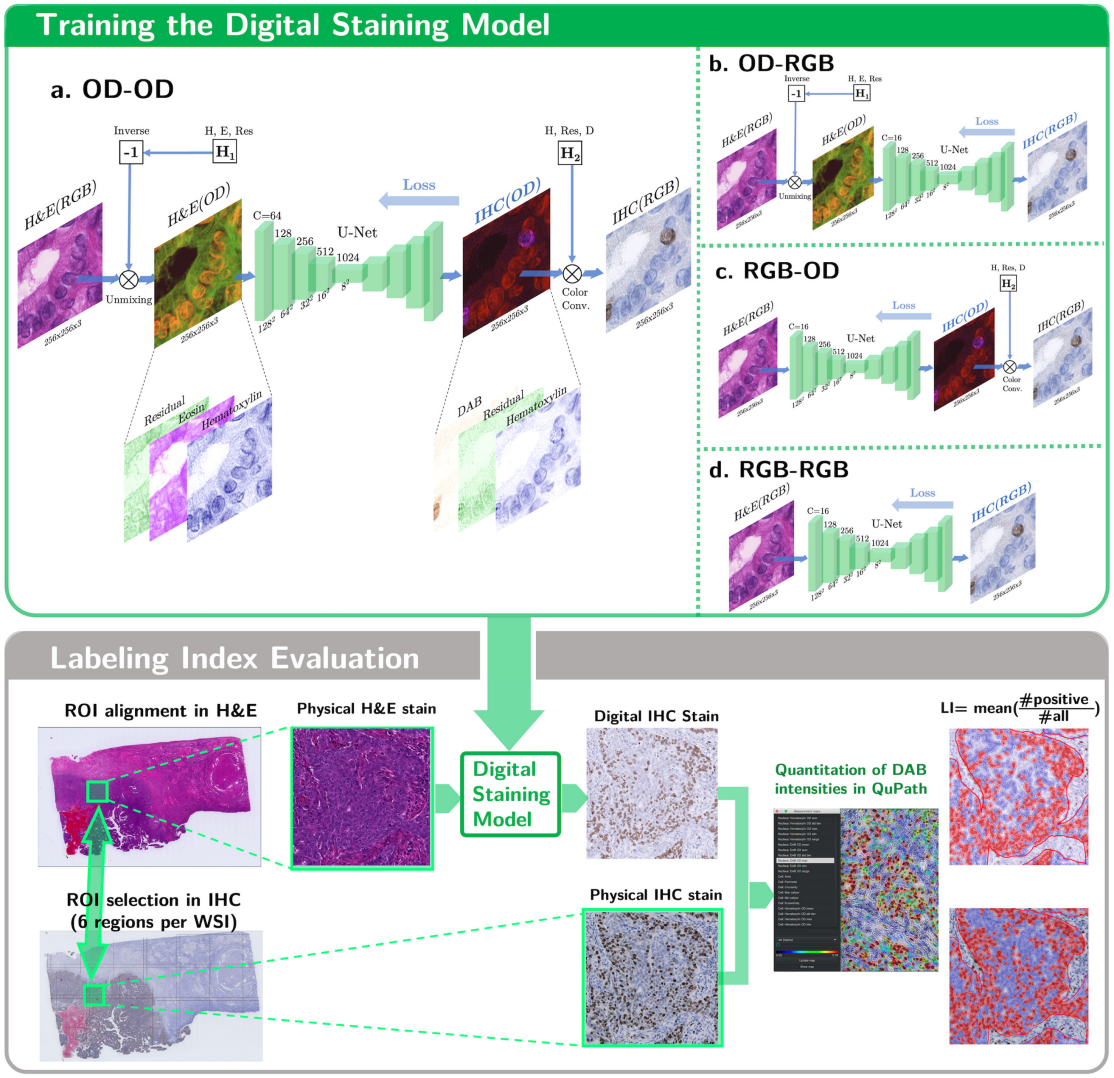
Overview of the methodology followed in this study. Training the U-Net with the physical IHC stain and the H&E stain in four color space combinations. The quality of the digital stains is evaluated with the correlation of LI.

We used the U-Net^12^ to directly predict the digital staining images in the OD or RGB space. Both models were trained in an end-to-end manner. The stain density maps were calculated from OD using the color unmixing technique (see Sec. 3.2). As for the physical processing of specimens, a section of a physical specimen was manually stained with H&E. After the physical specimen section was scanned and digitized as an H&E-staining WSI, we destained the very section and manually applied the IHC method on it. Finally, we scanned the IHC-stained physical specimen and used its WSI as the physical ground truth. After scanning and spatial registration, we extracted the OD of the stain’s IHC image using the color unmixing method. The image pairs of H&E-IHC were used to train the U-Net, as shown in Fig. 3. Four input–output color space combinations were used in the present study: OD–OD, RGB–RGB, RGB–OD, and OD–RGB, where OD images separate stains into individual channels.

For inference, we used the trained model to predict the OD image of the stains and convert the output OD to RGB or infer the RGB image of Ki-67 IHC staining directly. The matrix for color-unmixing was reused during OD–RGB output conversion.

### 3.2 Color Unmixing

The color unmixing method was used to separate the stains from an RGB image based on their absorption characteristics.^30^ Each pigment in the physically stained specimens has its own absorption coefficients for the R, G, and B lights. Thus it is assumed that the OD values (absorbance) of RGB components can be represented by a linear combination of the stain amounts.^31^

In the case of H&E staining, the OD image mentioned in the previous section consists of the stain density maps of H&E and the map of the background component. The term “stain density” represents the amount of stain estimated in each pixel. Since the absorption characteristic of the background is unknown and we have an image with only three channels, we approximately consider a residual component as the background.

Then the linear mixing relationship is given by

OD=cH, (1)

where $c=(C_{H},C_{E},C_{R})$ is a vector of the stain densities of hematoxylin and eosin and the intensity of the residual component, and $OD=({\mathrm{OD}}_{R},{\mathrm{OD}}_{G},{\mathrm{OD}}_{B})$ is a vector of OD values of R, G, and B components. H is a matrix of the absorption coefficients of hematoxylin and eosin and the coefficients for the residual component. H is sometimes called the stain matrix, and if $H_{1}$ is the stain matrix for H&E stain, we have

$$H=H_{1}=(\begin{array}{ccc} \epsilon_{\mathrm{HR}} & \epsilon_{\mathrm{HG}} & \epsilon_{\mathrm{HB}}\\ \epsilon_{\mathrm{ER}} & \epsilon_{\mathrm{EG}} & \epsilon_{\mathrm{EB}}\\ \epsilon_{\rho R} & \epsilon_{\rho G} & \epsilon_{\rho B} \end{array})=\begin{array}{c} \begin{array}{ccc} R & G & B \end{array}\\ \left(\begin{array}{ccc} 0.651 & 0.701 & 0.290\\ 0.070 & 0.991 & 0.110\\ -0.332 & -0.081 & 0.940 \end{array}\right) \end{array}\begin{array}{c} \text{hematoxylin}\\ \text{eosin}\\ \text{residual} \end{array},$$ (2)

where the absorption coefficient vectors of stain s, $\epsilon_{s}=(\epsilon_{sR},\epsilon_{sG},\epsilon_{sB})$ with s=H, E, and the residual coefficient vector, is obtained by the cross product $\epsilon_{\rho }=(\epsilon_{\rho R},\epsilon_{\rho G},\epsilon_{\rho B})=\epsilon_{H}\times \epsilon_{E}$. The exact definition of $\epsilon_{s}$ and its derivation are described at the end of this section. The actual values of matrix H given in Eq. (2) are obtained from the study conducted by Ruifrok et al.,^30^ although they may not be suitable for the slides used in the present study owing to the variations in the absorption characteristics caused by chemical conditions of the stain, staining time, and specimen transmittance.^32^ Quantitation and alleviation of such biases will be investigated in future studies.

Similarly, the OD image of H-DAB staining consists of the stain density maps of hematoxylin, DAB, and the map of the residual component. The eosin component in Eq. (2) is substituted with DAB. Thus the residual coefficient vector ${\epsilon^{\prime }}_{\rho }=(\epsilon_{\rho R}^{\prime },\epsilon_{\rho G}^{\prime },\epsilon_{\rho B}^{\prime })=\epsilon_{H}\times \epsilon_{\mathrm{DAB}}$. The stain matrix for H-DAB staining $H_{2}$ becomes

$$H=H_{2}=(\begin{array}{ccc} \epsilon_{\mathrm{HR}}^{\prime } & \epsilon_{\mathrm{HG}}^{\prime } & \epsilon_{\mathrm{HB}}^{\prime }\\ \epsilon_{\rho R}^{\prime } & \epsilon_{\rho G}^{\prime } & \epsilon_{\rho B}^{\prime }\\ \epsilon_{\mathrm{DR}}^{\prime } & \epsilon_{\mathrm{DG}}^{\prime } & \epsilon_{\mathrm{DB}}^{\prime } \end{array})=\begin{array}{c} \begin{array}{ccc} R & G & B \end{array}\\ \left(\begin{array}{ccc} 0.651 & 0.701 & 0.290\\ 0.633 & -0.713 & 0.302\\ 0.269 & 0.568 & 0.778 \end{array}\right) \end{array}\begin{array}{c} \text{hematoxylin}\\ \text{residual}\\ \mathrm{DAB} \end{array}.$$ (3)

Following the Beer–Lambert law,^33^ the pixel values $I=(I_{R},I_{G},I_{B})$, which represent the light intensities recorded by the sensor, were normalized by the maximum intensity $I_{0}=(I_{0R},I_{0\;G},I_{0B})$, which can be obtained from glass regions, and converted to the OD with an element-wise division followed by logarithm as shown in Eq. (4), where s=R,G,B.

$${\mathrm{OD}}_{s}=-\log_{10}(\frac{I_{s}}{I_{0\;s}}).$$ (4)

**Fig. 4:**
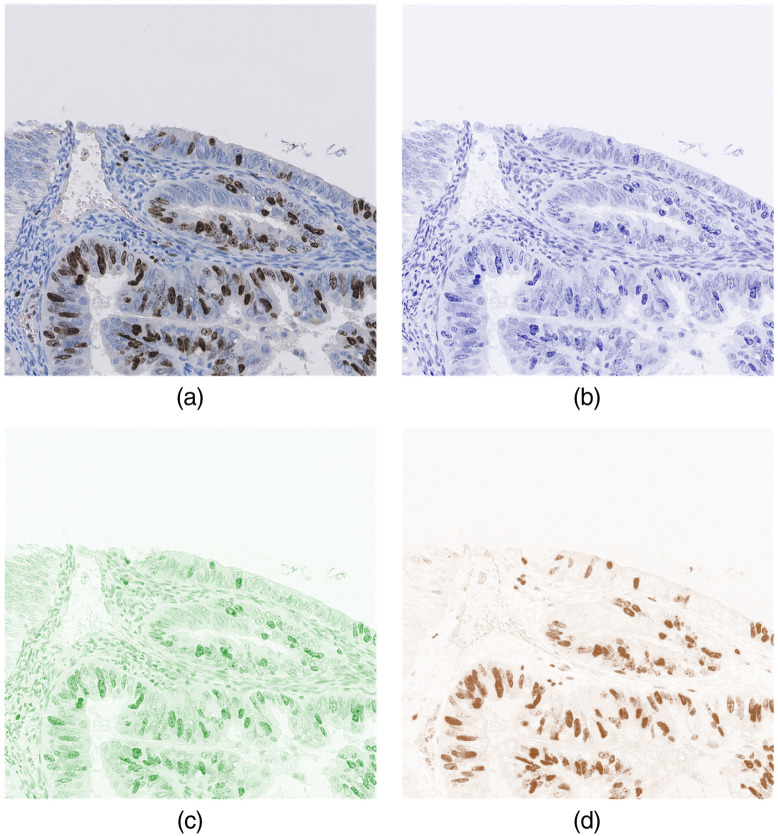
Color unmixing for physical IHC stains: (a) RGB input, (b) hematoxylin, (c) residual, and (d) DAB.

The density of each stain was calculated using the OD of the R, G, and B channels. For example, Eq. (5) shows the calculation of hematoxylin and eosin intensities from a H&E stained RGB image:

$$(C_{H},C_{E},C_{R})=({\mathrm{OD}}_{R},{\mathrm{OD}}_{G},{\mathrm{OD}}_{B})H_{1}^{-1},$$ (5)

where $H_{1}^{-1}$ is the inverse of the stain OD matrix $H_{1}$; $C_{H}$, $C_{E}$, and $C_{R}$ are the stain densities of hematoxylin, eosin, and residual components, respectively; $I_{R}$, $I_{G}$, and $I_{B}$ are the normalized intensities of the R, G, and B channels for each pixel, respectively. Figure 4 presents an example of a color-unmixed IHC stain, where the residual channel denotes the component orthogonal to the hematoxylin and DAB stains. The positive nuclei are clearly stained in the DAB channel, and the negative cells can be distinguished from the hematoxylin channel.

The exact definition of $\epsilon_{s}$ was derived according to the Beer–Lambert law. Let $A_{s\lambda }$ denote the absorbance of a sample that contains the material (stain) s for wavelength λ, ${\overset{˜}{\epsilon }}_{s\lambda }$ denotes the molar absorption coefficient of the material stain s, $c_{s}$ denotes the molar concentration of the stain, and l denotes the optical path length in the sample stain. Thus,

$$A_{s\lambda }={\overset{˜}{\epsilon }}_{s\lambda }c_{s}l.$$ (6)

If we neglect the scattering in the material, the intensity of the transmitted light with wavelength λ in a region purely stained with s, $I_{s\lambda }$ is given by

$$I_{s\lambda }=I_{0\lambda }10^{-A_{s\lambda }},$$ (7)

where $I_{0\lambda }$ is the light intensity incident into the material. Another approximation is to consider the wavelength λ only with R, G, and B color channels. The OD of a single-stained sample ${\mathrm{OD}}_{s}$ corresponds to the absorbance, ${\mathrm{OD}}_{s}=(A_{sR},A_{sG},A_{sB})$.

In WSI, $c_{s}l$ represents the amount of molecule in the effective cross section that corresponds to a single pixel. However, it is difficult to quantify the absolute amount of molecule and we do not need the absolute value of the material concentration. Now let us consider an arbitrary constant alpha for normalization. Then, we have $\epsilon_{s\lambda }=\alpha {\overset{˜}{\epsilon }}_{s\lambda }$ where $\epsilon_{s\lambda }$ denotes the relative absorption coefficient after normalization, and we define the relative amount of molecule $C_{s}=c_{s}l/\alpha$, whereas $A_{s\lambda }=\epsilon_{s\lambda }C_{s}$.

If we select pixels purely stained with the stain s and obtain ${\mathrm{OD}}_{s}$, we can determine the absorption coefficient vector $\epsilon_{s}=(\epsilon_{sR},\epsilon_{sG},\epsilon_{sB})$ by normalizing ${\mathrm{OD}}_{s}=(\epsilon_{sR},\epsilon_{sG},\epsilon_{sB})C_{s}$.

### 3.3 Spatial Alignment

Since we washed out the H&E stain and applied the H-DAB stain subsequently to visualize the Ki-67 positivity of the corresponding nuclear regions, the IHC and H&E images of one specimen have location misalignment caused by rescanning. Therefore, image registration of H&E and IHC WSIs was performed.

**Fig. 5:**
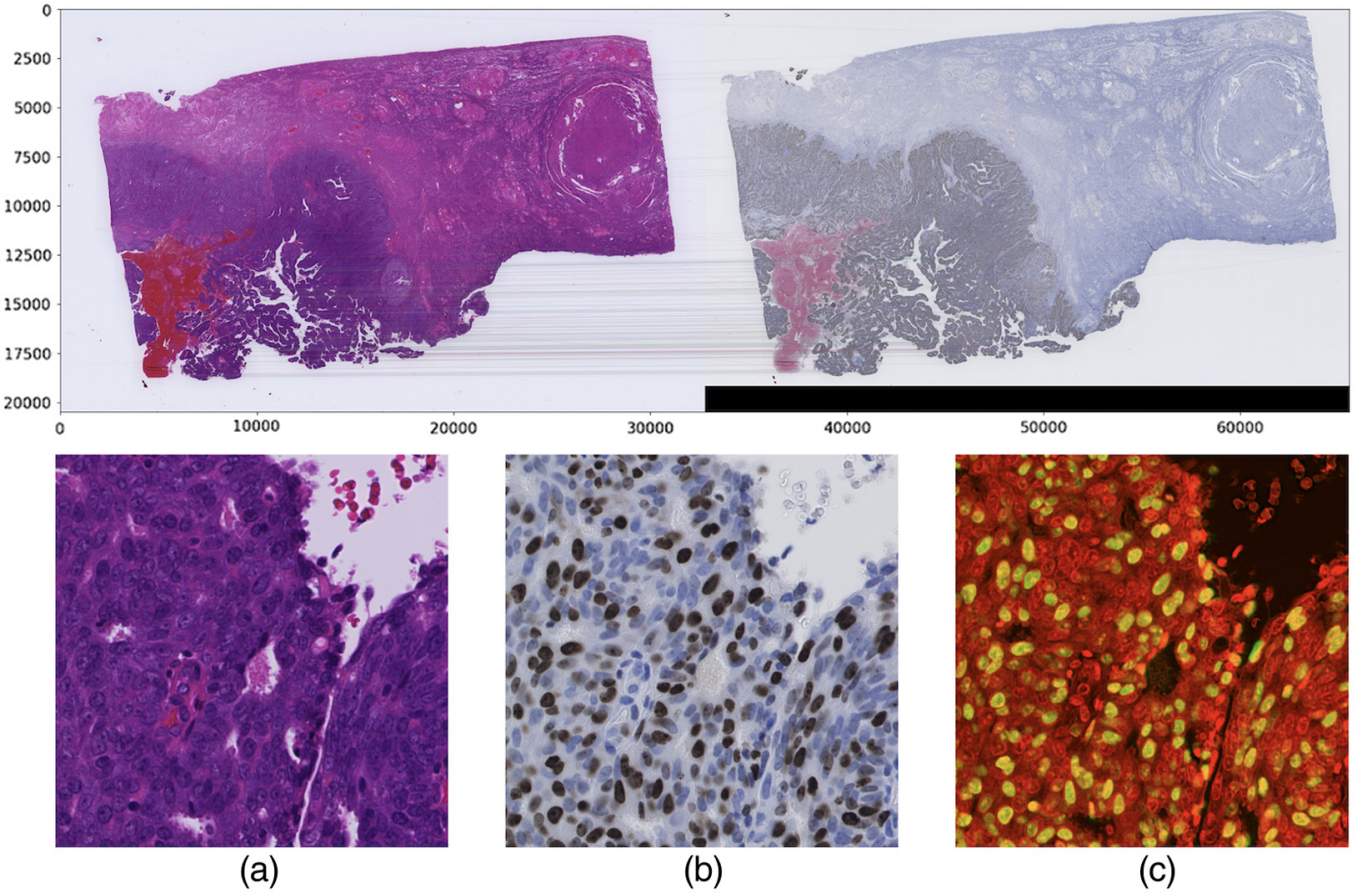
Global registration with ORB features and FLANN matching: (a) H&E, (b) IHC, and (c) superposition.

The registration was performed based on affine matrix estimation, the transformation from one biased image to the reference image. The implementation of Marzahl et al.,^34^ which used ORB features and FLANN matching,^35^ was applied in this study. Figure 5 shows an example of spatial alignment.

### 3.4 U-Net

**Fig. 6:**
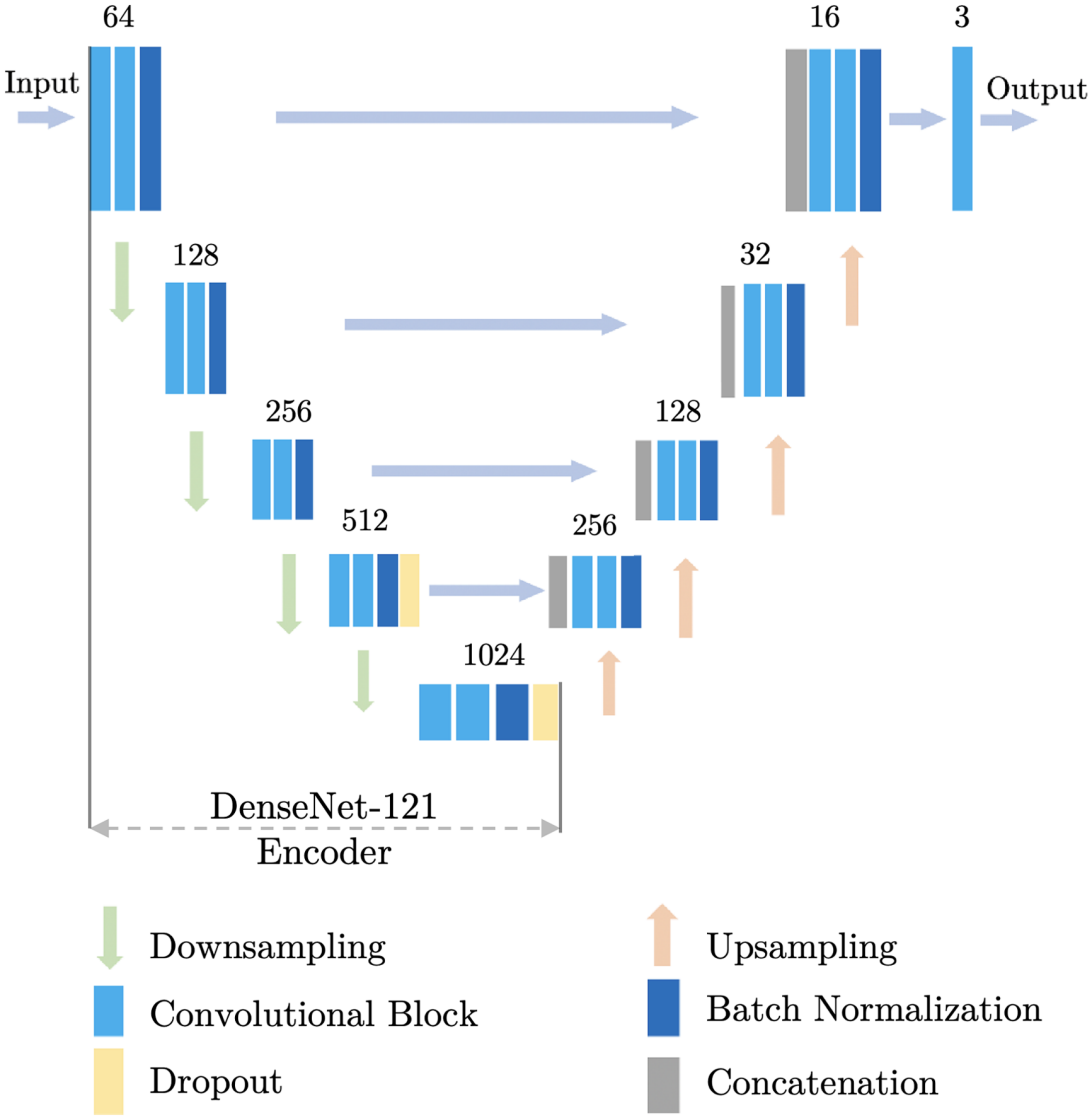
Architecture of the U-Net. We used the DenseNet-121 as its backbone.

Figure 6 shows the U-Net implementation. The numbers on the top of each convolutional module indicate the number of filters for the output convolutional layer in that module. The input shape of the network was 256×256 during training and arbitrary for inference. All filter sizes were 3×3, except for the output layer, which used 1×1 convolution to generate a three-channel output image. All downsampling and upsampling rates are 2×2. DenseNet-121 was used^36^ as the backbone of our network, which was pretrained on the ImageNet dataset.^37^ The models and pretrained weights were adapted from the implementation of segmentation models^38^ code base.

### 3.5 Training

The mean absolute error (MAE) was used as the loss function to train the U-Net, as defined by

loss(y,y^)=1N∑n=1N|yn−y^n|, (8)

where y^ and y correspond to the prediction and the ground truth, respectively. N is the number of pixels in a minibatch. As shown in Fig. 3, in all schemes, images in the color space of the U-Net’s input and output were prepared in advance, i.e., we calculated and backpropagated the loss with the output stain density map and a precomputed ground truth if the U-Net predicts OD rather than computing the loss with the RGB IHC stain after postprocessing. Identical $H_{1}$ and $H_{2}$ were used in all training and predictions.

To validate the generalization ability of our method, we designed two different schemes for training and validation.

**Fig. 7:**
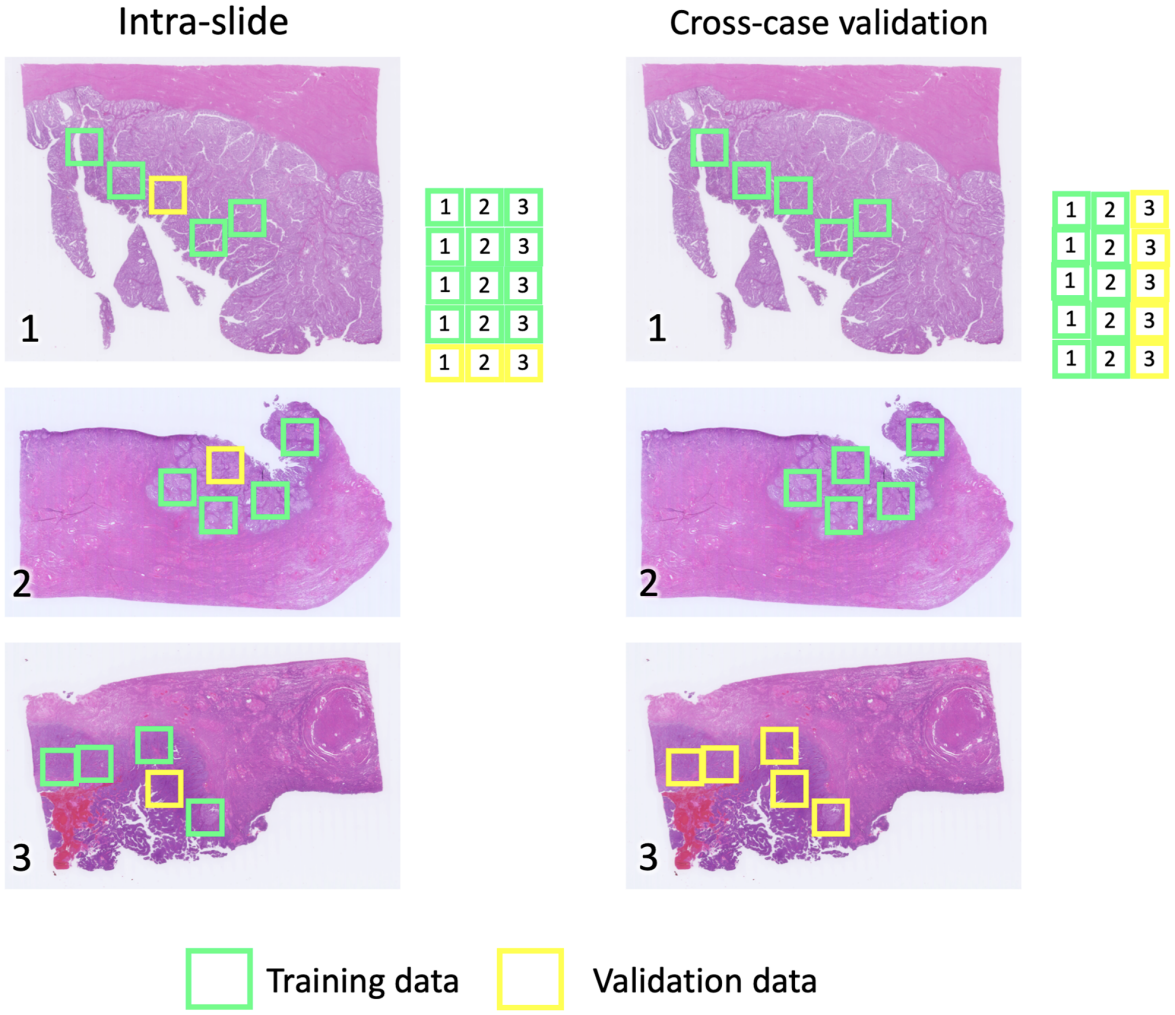
Training and validation schemes.

The intraslide training and validation scheme included all WSIs in the training set; the randomly sampled regions in each case were used for validation. As shown in Fig. 7, the green grids represent the data shards used for training, whereas the yellow grids represent data for validation. Intraslide inference can be used to generate digital staining of tiles when annotation and IHC staining of other tiles in the same tissue are available. This scheme was used frequently in previous reports. However, the similarities in tissue structure and staining condition in the intraslide scheme may introduce biases, thereby preventing its generalization to other cases. This gap is experimentally presented in Sec. 5.3.

The cross-case validation scheme was used to test the model’s generalization ability across the cases. That is, we took sixteen cases in each grade from the dataset for training and left three cases in each grade for validation. In this sixfold validation, no information from any regions in the testing cases was involved in training, and the effectiveness of the models’ cross-case prediction could be qualitatively shown.

## 4 Experiment

### 4.1 Hardware and Software

As Table 1 shows, we used TensorFlow 2.0^39^ as the basic framework for neural network construction and data processing. QuPath^40^ was adopted as a third-party tool for annotation and evaluation. All experiments were performed on the Nvidia DGX workstation with quad V100 GPUs, each with 32 GB of memory. A batch size of 64 was used for each GPU. The Adam^41^ optimizer’s base learning rate of $2.5\times 10^{-4}$ was scaled by the number of GPUs operating in parallel.^42^ Four GPUs were utilized for the training. The hyperparameters of the Adam optimizer were $\beta_{1}=0.9$, $\beta_{2}=0.999$, and $\epsilon =1\times 10^{-7}$. No weight decay was used. We used a U-Net with a DenseNet-121 backbone, i.e., DenseNet-121-based encoder layers, and the model was trained for 50 epochs, taking approximately 11 h. All inference results were obtained with models at epoch 50. The test results of the cross-case models were generated using the model of the corresponding fold. The identical U-Net architecture and backbone for the generator were used while training the GAN-based Pix2Pix and Cycle-GAN models. The models were trained using the same step number.

**Table 1:** Summary of the experiment settings.

Model	U-Net	Pix2Pix	Cycle-GAN
Optimizer	Adam, $\mathrm{LR}=1\times 10^{-3}$ $\beta_{1}=0.9$, $\beta_{2}=0.999$	Adam, $\mathrm{LR}=2\times 10^{-4}$ $\beta_{1}=0.5$, $\beta_{2}=0.999$	Adam, $\mathrm{LR}=1\times 10^{-4}$ $\beta_{1}=0.5$, $\beta_{2}=0.999$
Loss	Eq. (8)	Eq. (9), λ=100	Eq. (10), $\lambda_{1}=5$, $\lambda_{2}=1$
Generator	U-Net with DenseNet-121 encoder backbone		
Machine	DGX Station, Quad Nvidia V100		
Framework	TensorFlow 2.3.1		
Batch size	256		
Epochs	50		

The objective of Pix2Pix is shown in the following equation:

$$G_{\mathrm{Pix}2\mathrm{Pix}}^{*}=\arg\;\underset{G}{\min}\;\underset{D}{\max}\{L_{\mathrm{GAN}}(G,D)+\lambda L_{L1}(G)\},$$ (9)

where G(x) is the generated IHC patch, y is the ground-truth IHC patch, x is the input H&E patch, G(·) is the generator, D(·) is the discriminator, $L_{\mathrm{GAN}}(G,D)=E_{x,y}[{\left\|D(y)-D(G(x))\right\|}_{2}]$ is the adversarial loss term, $L_{L1}(G)=E_{x,y}[{\left\|y-G(x)\right\|}_{1}]$ is the L1 loss term, and E[·] is the mathematical expectation calculated by averaging a minibatch. ${\left\|\cdot \right\|}_{2}$ and ${\left\|\cdot \right\|}_{1}$ are L2 norm and L1 norm, respectively. The weight factor λ=100.^18^ Training the Pix2Pix for 50 epochs took ∼14  h.

The objective of Cycle-GAN is shown in the following equation:

$${G^{*}}_{\text{CycleGAN}}=\arg\;\underset{G,F}{\min}\;\underset{D_{x},D_{y}}{\max}\{L_{\mathrm{GAN}}(G,D_{y},x,y)+L_{\mathrm{GAN}}(F,D_{x},y,x)+\lambda_{1}L_{\mathrm{cyc}}(G,F)+\lambda_{2}L_{\mathrm{id}}(G,F)\},$$ (10)

where x is the H&E patch, y is the ground-truth IHC patch, G(·) is the generator converting an H&E patch to IHC, F(·) is the generator converting an IHC patch to H&E, $D_{y}$ is the discriminator for generated IHC patches, and $D_{x}$ is the discriminator for generated H&E patches. $L_{\mathrm{GAN}}(G,D_{y},x,y)=E_{x,y}[{\left\|D_{y}(y)-D_{y}(G(x))\right\|}_{2}]$ is the adversarial loss, $L_{\mathrm{cyc}}(G,F)=E_{x}[{\left\|F(G(x))-x\right\|}_{1}]+E_{y}[{\left\|G(F(y))-y\right\|}_{1}]$ is the cycle consistency loss such that F(G(x)), the output of the IHC-H&E generator, approximates the ground-truth H&E image x and vice versa for G(F(y)), the output of the H&E-IHC generator. $L_{\mathrm{id}}(G,F)=E_{y}[{\left\|G(y)-y\right\|}_{1}]+E_{x}[{\left\|F(x)-x\right\|}_{1}]$ is the identity loss such that the H&E-IHC generator G(·) does not change an IHC input y and vice versa for the IHC-H&E generator F(·). The weight factors $\lambda_{1}=5$, $\lambda_{2}=1$.^23^

RGB–RGB color space was used to train the Cycle-GAN models owing to the heavy computation. The learning rate of Adam was set to $2\times 10^{-4}$ for Pix2Pix and $1\times 10^{-4}$ for Cycle-GAN. $\beta_{1}$ was set to 0.5. The remaining hyperparameters of the GAN-based methods are identical to the proposed method. Training the Cycle-GAN for 50 epochs took ∼45  h. On average, inference of the U-Net generator in all models required 2.9 s with CPU and 1.5 s with GPU for a 2048×2048 tile in the test set.

### 4.2 Dataset

#### 4.2.1 Pathology specimens

To acquire original pathological data of paired H&E and IHC stains, we used specimens of UCEC diagnosed at the Shinshu University Hospital. Fifty-seven cases classified as G1, G2, and G3 according to the International Federation of Gynecology and Obstetrics (FIGO) classifications^43^ were used in this study. Each grade comprised 19 cases-specimens. The H&E stained specimens were decolorized after scanning, and the IHC reaction for Ki-67 was performed on the same specimens. This process reveals positive reactions of the nuclei in the IHC and shows the fundamental morphological and texture features in the H&E specimens. The IHC reaction was performed using the Novolink Polymer method (Leica Biosystems, Nussloch, Germany). The primary antibody against the Ki-67 protein (clone: MIB-1, Dako, Santa Clara, California, USA) was allowed to react at room temperature for 1 h. The IHC reaction products were visualized by a DAB substrate chromogen with deep brown. Ki-67 negative nuclei were stained blue with Mayer’s hematoxylin, thereby yielding high visual contrast.

Both H&E-stained and IHC-stained specimens were scanned using a whole slide scanner (NanoZoomer 2.0-HT, Hamamatsu Photonics Corp., Shizuoka, Japan) with a 40× objective lens (pixel pitch = 0.2263  μm). The WSIs were aligned subsequently. Thus, 57 pairs of H&E-stained and IHC-stained WSIs of the physical specimens were obtained.

#### 4.2.2 Sampling and preprocessing

**Fig. 8:**
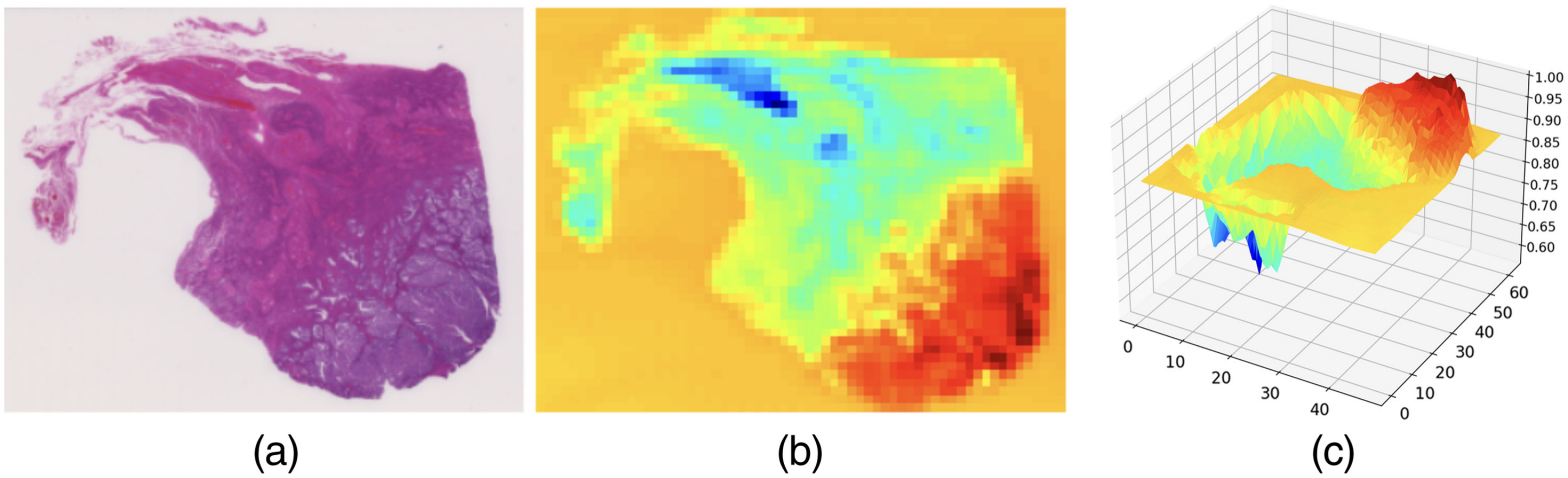
Sampling of the ROIs with the blue ratio. (a) H&E image; (b) quantitation of blue ratio in the 2048×2048 grids; and (c) surface plot showing the peak of blue ratio, corresponding to the tumor.

Manual registration of all 57 cases was laborious and infeasible. We used affine matrix estimation from ORB features and FLANN matching instead. The window size for keypoint extraction was set to 64×64, and the maximum number of features was set to 131,072. Registration was performed on WSIs downsampled to 32,768 pixels of width. The registration error was evaluated by comparing the MAE of the x and y coordinates among ninety landmark points manually set in nine cases. The average registration error was Δx=1.4  μm (6.4 pixels) and Δy=0.9  μm (3.8 pixels). The error with that of manual registration yielding Δx=1.8  μm and Δy=0.7  μm for the same images. Thus automatic registration was considered acceptable (Fig. 8).

To extract the ROIs and build the dataset, tiles with the size of 2048×2048 were sampled according to the blue ratio of the downsampled WSIs. The regions with a higher blue ratio were considered to have concentrated tumor cells stained with hematoxylin. These regions are considered suitable for training.^44^ After preprocessing, 7370 samples with the size of 2048×2048  pixels were extracted from the 57 pairs of WSIs. We randomly selected six samples from each WSI in advance and used them for testing. As a result, we have 7028 sets of 2048×2048 H&E-IHC tile pairs in OD and RGB color spaces for training and validation and 342 sets for testing. The tile pairs in the training and validation splits were selected randomly during runtime with a fixed random seed. Those tiles were cropped to 256×256  pixels in the training phase.

### 4.3 Evaluation Metrics

#### 4.3.1 Labeling index

We evaluate the Pearson correlation of Ki-67 LI calculated from the digital staining results and the corresponding physical stains. Ki-67 LI is the proportion of Ki-67-positive cells in a tumor region. The calculation of LI is shown in Eq. (11), where $N_{\mathrm{Ki}-67}^{\left(+\right)}$ is the number of positive nuclei in the tumor regions and $N_{\mathrm{Ki}-67}^{\left(-\right)}$ is the number of negative nuclei:

$$\mathrm{LI}=\frac{N_{\mathrm{Ki}-67}^{\left(+\right)}}{N_{\mathrm{Ki}-67}^{\left(+\right)}+N_{\mathrm{Ki}-67}^{\left(-\right)}}.$$ (11)

As shown in Fig. 3, six patches excluded from training and validation sets were sampled from each WSI. Identical parameters for postprocessing were set in the QuPath quantitation software for nucleus counting in all experiments. The derived labeling indices may vary according to the parameter settings and the selection of evaluation regions.

#### 4.3.2 Image similarity

The image similarity metrics that are commonly used in image processing tasks were evaluated to compare the proposed method with the baseline comprehensively. We report the peak signal-to-noise ratio (PSNR) and the structural similarity index measure (SSIM)^45^ of the digital stain. Let y^ and y denote the prediction and the ground truth images, respectively. The PSNR is defined in the following equation:

PSNR(y^,y)=20·log10(max(y^)‖y^−y‖22), (12)

where max(·) is the maximum value function. The SSIM is defined in the following equation:

SSIM(y^,y)=(2μy^μy+c1)(2σy^y+c2)(μy^2+μy2+c1)(σy^2+σy2+c2), (13)

where $\mu_{\hat{y}}$ and $\mu_{y}$ are the pixel sample mean, $\sigma_{\hat{y}}$ and $\sigma_{y}$ are the standard deviation, and $\sigma_{\hat{y}y}$ is the cross correlation of y^ and y. $c_{1}$ and $c_{2}$ are small factors for numerical stabilization. Because registration errors exist in the preprocessing of our dataset, there are location misalignments between the H&E image and the IHC image. When evaluating the similarity metrics between the digital IHC stain, which is generated from physical H&E and the physical IHC stain, the translation sensitivity of PSNR and SSIM would result in lower, biased scores. Therefore, we also report the complex wavelet SSIM (CW-SSIM),^46^ which computes the similarity of images in the frequency domain and alleviates the effect of registration errors. We computed the average of CW-SSIM between the channels of the digital IHC staining image and the physical IHC staining image.

## 5 Result

### 5.1 Visual Result

**Fig. 9:**
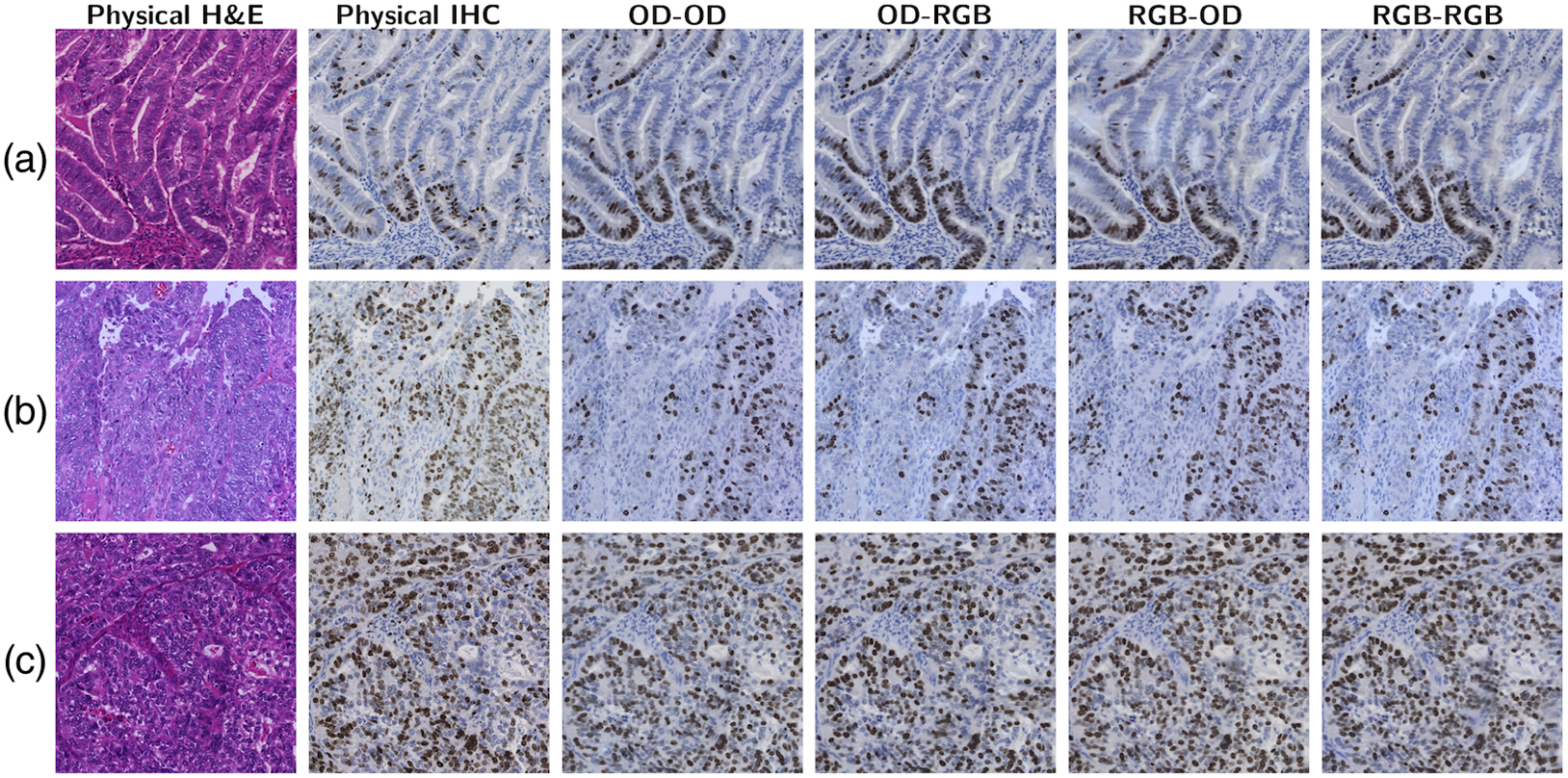
Visual results of U-Net under the cross-case scheme. (a)–(c): G1, G2, and G3.

**Fig. 10:**
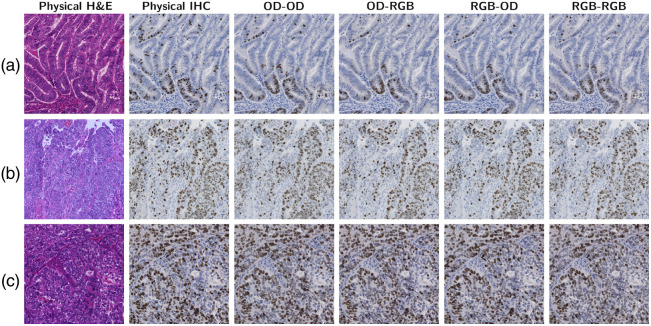
Visual results of U-Net under the intraslide scheme. (a)–(c): G1, G2, and G3.

**Fig. 11:**
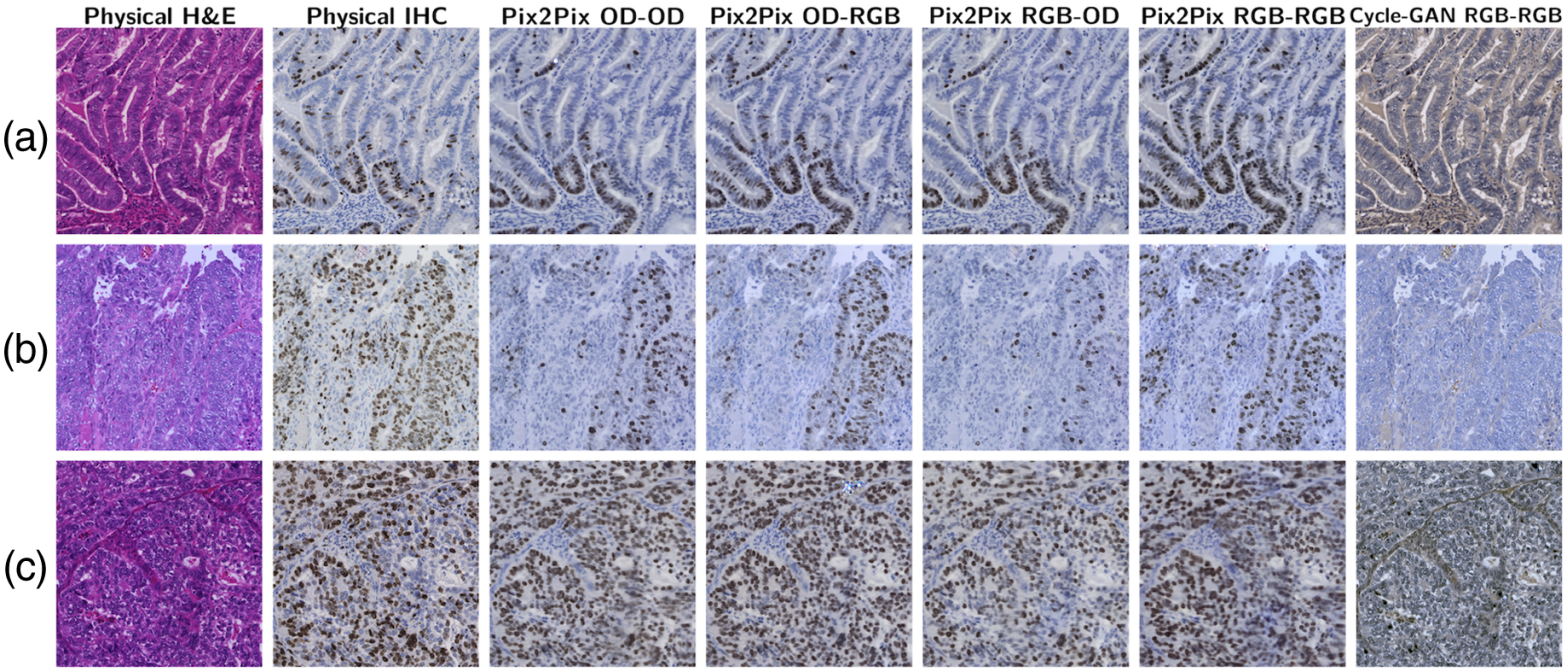
Visual results of GAN-based models under the cross-case scheme. (a)–(c): G1, G2, and G3.

**Fig. 12:**
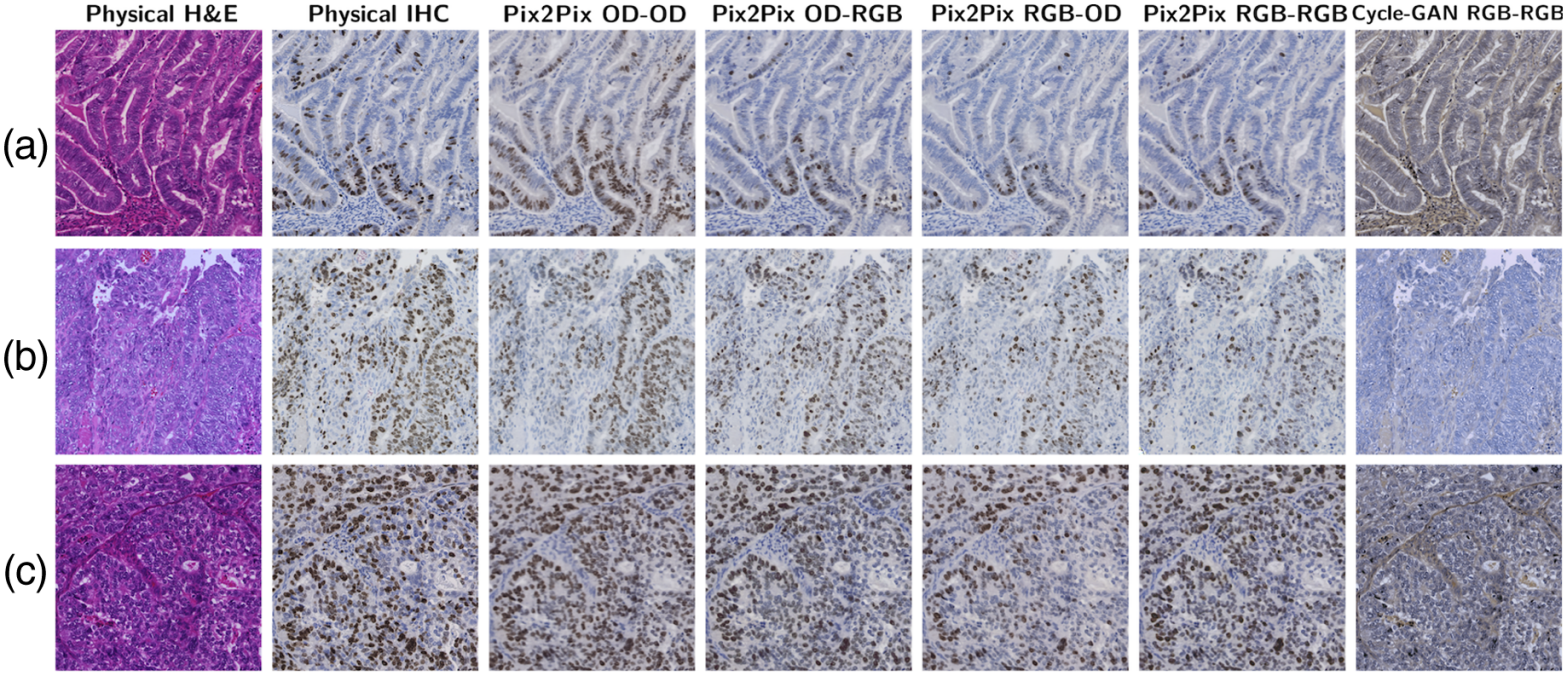
Visual results of GAN-based models under the intraslide scheme. (a)–(c): G1, G2, and G3.

We report the results using OD–OD, RGB–RGB, OD–RGB, and RGB–OD color spaces under intraslide and cross-case schemes. Figures 9 and 10 show the tiles of the H&E specimens, corresponding physical IHC stains, and the digital stains generated from the U-Net. Figures 11 and 12 show the results of GAN-based models. The size of the test images was 2048×2048. The intraslide models generated results with loyal colors and precise Ki-67 positivity predictions. In contrast, the cross-case models exhibited artifacts, such as local blurring and color variations. The distribution of the Ki-67-positive nuclei was correlated with the physical staining in general. However, the advantages and disadvantages of color space combinations could not be determined qualitatively. Pix2Pix generates images with acceptable colors and positivity. The Cycle-GAN model failed to demonstrate a meaningful Ki-67-positive cell distribution even under the intraslide scheme. Moreover, the color of the generated images differed significantly from the ground truth.

### 5.2 Similarity Metrics

Table 2 presents the pixel level PSNR, SSIM, and CW-SSIM of U-Net-based, end-to-end models with different input/output color space combinations for intraslide and cross-case experiments. “O” and “R” correspond to the OD space and RGB space, respectively. For example, “OR” means the model uses OD input and RGB output to train the generator. The difference between the color space combinations was not prominent in general. Slightly higher scores of RGB–RGB metric were indicated by the potentially better color and structural fidelity of the result images; however, nucleus-level comparison necessitates further evaluations. In terms of the image similarity metrics, the Pix2Pix model achieved scores that were comparable with those of the U-Net-based models. We only report the results of Cycle-GAN with RGB–RGB generators; the experiments for color space combinations other than RGB–RGB were not conducted due to obvious visual and quantitative inferiority.

**Table 2:** Similarity metrics of different models with various color space combinations.

Model	Color	Intraslide			Cross-case		
		PSNR ↑	SSIM ↑	CW-SSIM ↑	PSNR ↑	SSIM ↑	CW-SSIM ↑
U-Net	OO	19.68	0.53	0.78	16.35	0.40	0.65
	OR	19.93	0.54	0.79	16.34	0.39	0.65
	RO	19.37	0.51	0.77	16.33	0.39	0.65
	RR	19.67	0.53	0.78	16.50	0.40	0.66
Pix2Pix	OO	18.05	0.46	0.70	16.61	0.41	0.64
	OR	19.44	0.50	0.76	16.61	0.41	0.64
	RO	18.70	0.50	0.72	16.58	0.41	0.64
	RR	19.26	0.50	0.75	16.31	0.40	0.64
Cycle-GAN	RR	15.07	0.33	0.60	14.76	0.31	0.60

### 5.3 Quantitation of Labeling Index

**Fig. 13:**
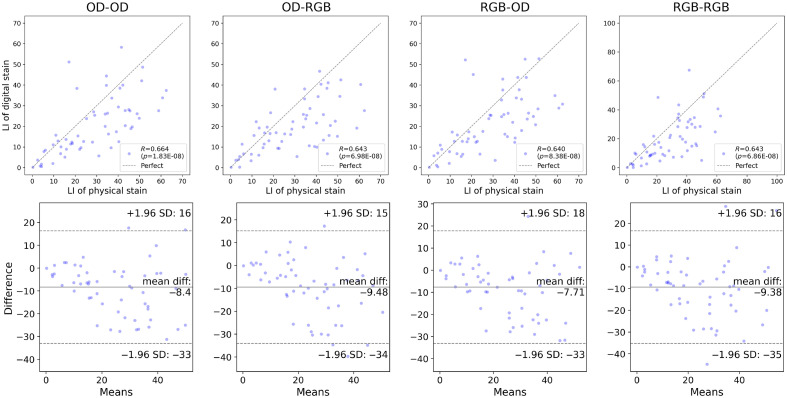
Pearson correlation and Bland–Altman plots of the LI derived using U-Net, calculated from the digital and physical stains, cross-case validation.

**Fig. 14:**
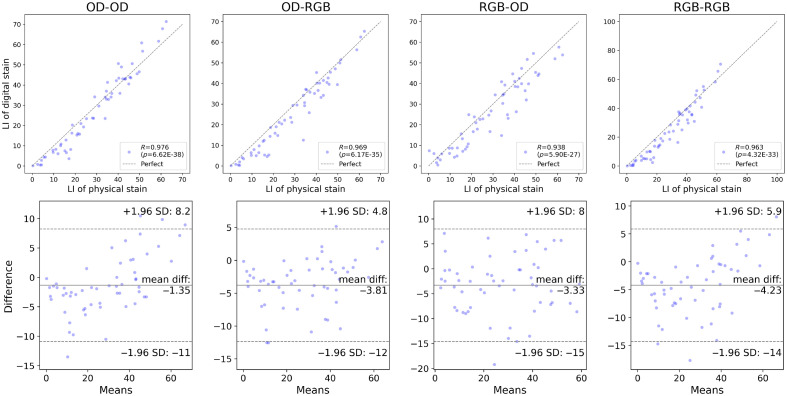
Pearson correlation and Bland–Altman plots of the LI derived using U-Net, calculated from the digital and physical stains, intraslide validation.

**Fig. 15:**
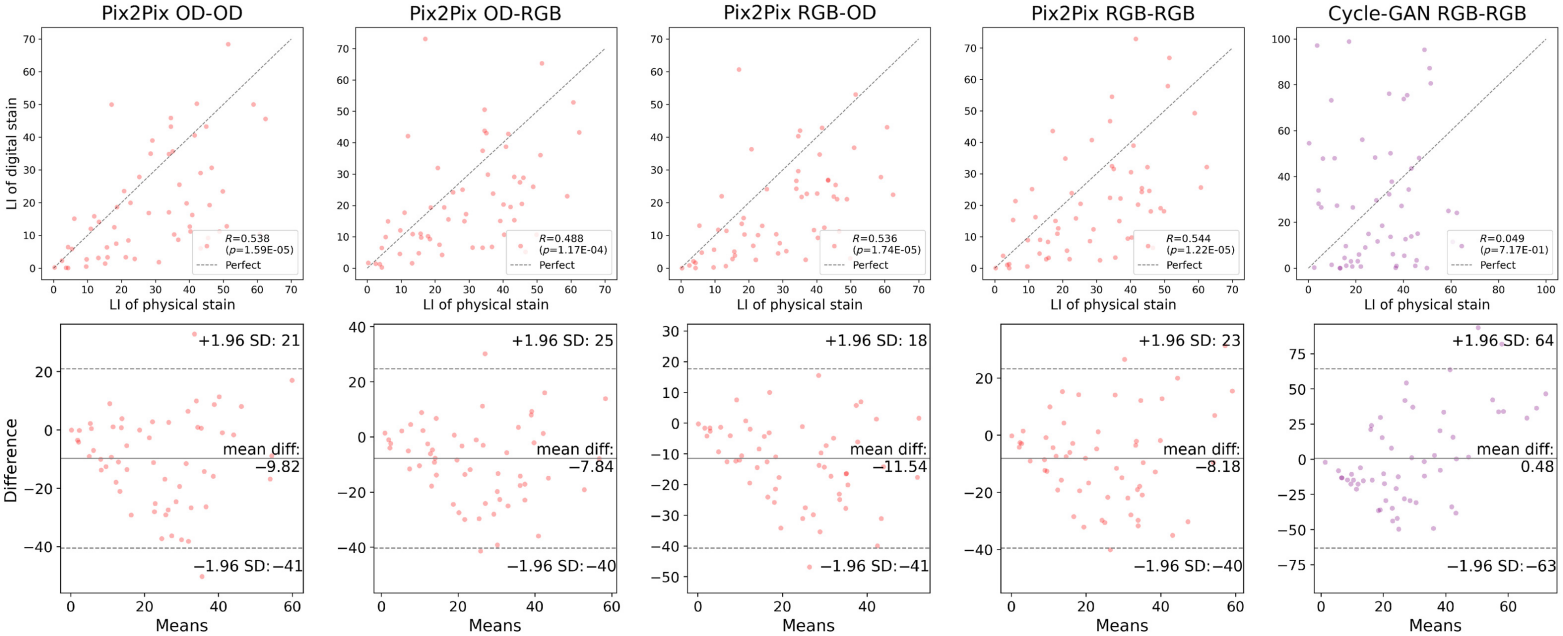
Pearson correlation and Bland–Altman plots of the LIs of the GAN models, calculated from the digital and physical stains, cross-case validation.

**Fig. 16:**
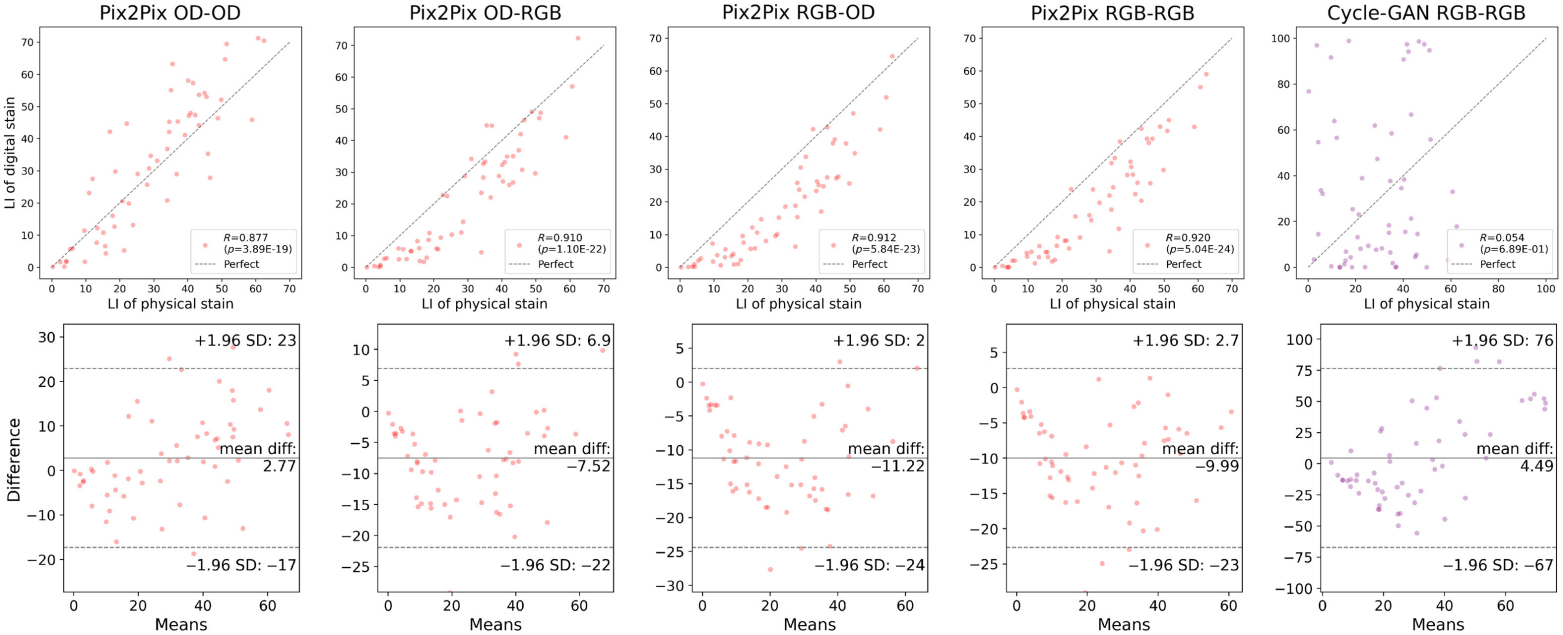
Pearson correlation and Bland–Altman plots of the LIs of the GAN models, calculated from the digital and physical stains, intraslide validation.

Figures 13 and 14 present the Pearson correlation and Bland–Altman plots of the LI derived using the U-Net, respectively. The U-Net yielded a correlation stronger than R=0.90 with statistical significance when the grade of each case was not addressed, i.e., according to the intraslide scheme. However, the digital staining models trained with the cross-case scheme were not equally correlated with the physical IHC staining. The differences in the mean values were also quantitated and visualized with Bland–Altman plots. The statistical analysis results are summarized in Tables 3 and 4, wherein agreement means there is no significant difference between the mean value of the LIs of the digital and physical stains according to a two-sided t-test. The p-values of the Pearson correlation and two-sided t-test were measured. The output of the model was considered consistent with the physical stain when the t-test revealed an insignificant difference (p>0.05). We also quantitatively showed the error with the MAE of the LI. The results of the intraslide models were consistent with the physical stain, indicating a strong correlation. Although weaker, a correlation was also observed in the CCV models, indicating the utility of digital Ki-67 staining in the future after considerable improvement in the technology. The Bland–Altman plots revealed negative biases, indicating the necessity to alleviate false negatives, especially in high-grade cases. As shown in Figs. 15 and 16, the Pearson correlation of Pix2Pix and Cycle-GAN failed to outperform the U-Net under any training scheme.

**Table 3:** Statistical evaluation results, intraslide. $p<10^{-9}$ are shown as 0.

Model	Color	Pearson correlation		Mean LI bias		LI MAE ↓
		Coeff. ↑	p-value	Bias ↓	Agreement (p)	
U-Net	OO	**0.98**	0	**−1.35**	✓(0.69)	**4.1**
	OR	0.97	0	−3.81	✓(0.23)	4.3
	RO	0.94	0	−3.33	✓(0.28)	5.3
	RR	0.96	0	−4.23	✓(0.20)	5.3
Pix2Pix	OO	0.88	0	2.77	✓(0.43)	8.2
	OR	0.91	0	−7.52	✗(0.02)	8.5
	RO	0.91	0	−11.22	✗($3\times 10^{-4}$)	11.4
	RR	0.92	0	−9.99	✗($2\times 10^{-3}$)	10.1
Cycle-GAN	RR	0.05	0.7	4.49	✓(0.37)	30.0

Note: bold values represent the best result obtained for each metric.

**Table 4:** Statistical evaluation results, cross-case validation.

Model	Color	Pearson correlation		Mean LI bias		LI MAE ↓
		Coeff. ↑	p-value	Bias ↓	Agreement (p)	
U-Net	OO	**0.66**	$2\times 10^{-8}$	−8.40	✗($4\times 10^{-3}$)	11.8
	OR	0.64	$7\times 10^{-8}$	−9.48	✗($6\times 10^{-3}$)	11.8
	RO	0.64	$8\times 10^{-8}$	**−7.71**	✗($8\times 10^{-3}$)	**11.4**
	RR	0.64	$7\times 10^{-8}$	−9.38	✗($2\times 10^{-3}$)	12.3
Pix2Pix	OO	0.54	$2\times 10^{-5}$	−9.82	✗($2\times 10^{-3}$)	13.9
	OR	0.49	$1\times 10^{-4}$	−7.84	✗($1\times 10^{-2}$)	14.0
	RO	0.54	$2\times 10^{-5}$	−11.54	✗($1\times 10^{-4}$)	14.8
	RR	0.54	$1\times 10^{-5}$	−8.18	✗($1\times 10^{-2}$)	14.7
Cycle-GAN	RR	0.05	0.72	0.48	✓(0.91)	26.4

Note: bold values represent the best result obtained for each metric.

## 6 Discussion

The two main features of the proposed method are as follows. First, it intuitively revealed the positivity of cells intuitively on the resultant images, and the resolution of the generated digital stain was higher than that of the FCN-based method.^26^ The generated digital stains, wherein the textures of chromatin and stromal tissues are preserved, were more intelligible for pathologists. Second, the proposed method utilized the color unmixing method to separate the stains, thereby enabling the direct supervision of the Ki-67 positive regions in the DAB channel without necessitating the manual annotation of each nucleus. The preprocessing procedure based on color unmixing could facilitate the explicit extraction of Ki-67-positive nuclei, even from low-quality stains; the methods based on generative models do not focus on the semantics and harm the explainability of the model.

OD–OD inference yielded the highest correlation with the ground truth in the intraslide and cross-case schemes. It was presumed that the color difference of the output image affects the results of nuclei quantification, as the effective features for distinguishing positive nuclei are mainly textural and chromatic. Thus calculating the OD for the input and output can provide clearer supervision of positive pixels and address the variation of each stain separately. Also unmixing the stain channels will facilitate stain intensity adjustment and color normalization of WSIs. Using such per-stain labels might contribute to the cross-case generalization of the digital staining models.

The primary limitation of the current method is the difficulty in generalizing the high prediction precision to cross-case scenarios. This generalization gap may be attributed to the color differences and redundant global information. With the stains in WSIs separated into OD channels, it would be feasible to normalize the staining intensities in training, which is a part of our future work. The U-Net accepts input images containing a tissue region rather than a single nucleus and involves global characteristics, such as glandular structures and specific patterns of cell swarm, during training. Such features vary in each case and can hinder the cross-case inference of our models.

There have been previous studies on digital staining; however, practical evaluations for clinical applications have not been conducted. The evaluation has primarily relied on assessing the visual similarity between digital images and their physically stained counterparts. As the purpose of IHC is to evaluate protein expression, it is meaningless unless the performance of digital IHC is assessed with a clinically relevant index. The result of evaluating with LI is quite valuable in positioning the potential for clinical application. The OD of DAB is also utilized as a diagnostic index and is an issue of future challenge.

Two evaluation schemes were compared in this study. Naturally, none of the schemes used the same patches for training and validation data. However, in the intraslide scheme, the training and validation sets included the images from the same slide. A high correlation was observed in that case, whereas the correlation decreased remarkably when the training and validation data were separated by case. It should be noted that only a single slide from each case was used in this study. If multiple slides are created for a single case, even though they are different slides, they should be considered as intraslide data and treated accordingly.

The trending GAN-based domain transfer models, particularly Cycle-GAN and Pix2Pix, did not exhibit superiority in the nucleus quantitation task, though competitive pixel-level image similarity metrics were observed with the use of Pix2Pix. The Cycle-GAN failed to yield a correlated result due to the lack of effective fidelity supervision, such as L1 loss. A cross-case generalization gap was also observed in the Pix2Pix model, and its nucleus-level LI correlation was even lower than that of the RGB–RGB U-Net baseline. Thus methods with direct fidelity loss, like L1, are preferred over generative frameworks.

It is essential to refer to the CCV evaluation and strive for further improvement to facilitate the wider application of deep learning in clinical practices. Previous studies have not specified whether to use the CCV or intraslide scheme. However, it is crucial to explicitly state the training scheme used, as it can lead to a significant difference in the results.

On the other hand, there might be use cases resembling the intraslide scheme, although it is currently challenging and requires ingenuity in the case of digital staining. In such unique use cases, the results obtained from the intraslide evaluation can serve as a reference.

## 7 Conclusion

We propose a digital staining model that utilizes the OD of stains and converts an image of a hematoxylin-eosin stain to its hematoxylin-DAB stain counterpart. We examined the correlation between the digital stain and the physical stain with the Ki-67 LI, a diagnostic metric widely used in clinical practices for cell proliferation assessment. The algorithm was evaluated with 57 WSIs for cell proliferation assessment, and the results indicate that the U-Net can generate a real-like digital stain that fairly correlates with the ground truth. We tested color space combinations of OD and RGB color spaces. Conversion from OD of H&E to OD of IHC yielded the highest correlation compared with other choices.

Correlation and bias analysis revealed a tendency toward a lower prediction of LI value and false negatives. A comparison of the CCV and intraslide training schemes revealed that the correlation coefficients of LI were 0.66 and 0.98 for the CCV and intraslide schemes, respectively. The accuracy of CCV must be enhanced to enable its application in digital staining technology; namely, the model’s generalizability across the cases must be improved. In some other publications, it is unclear whether the evaluation is conducted across cases or not. This study demonstrated a high correlation for the intraslide scheme but a considerably lower correlation for the CCV scheme. Thus the agreement of diagnostic metrics, such as the LI, should be evaluated via case-based cross validation or clearly stated in the report. Although the current model could not yield a diagnostically precise digital stain for every specimen, a significant correlation was observed even during cross-case evaluation. Digital stains will assist pathologists in identifying the expression of Ki-67 in the specimens and determining the malignancy of neoplasms.

## Data Availability

The programs are available on https://github.com/jic-titech/ki67, where the readers can also access raw data, including the evaluation images and the model weights for reproducing experiment results presented in this paper.
